# Supplementary material for: KAT6A mutations in Arboleda-Tham syndrome drive epigenetic regulation of posterior HOXC cluster
Source: Hum Genet. 2023 Oct 20;142(12):1705–20. doi: 10.1007/s00439-023-02608-3 (PMC10676314; doi:10.1007/s00439-023-02608-3)
Supplement: Supplementary file 2 — Supplementary file2 (PDF 1374 KB) [file 439_2023_2608_MOESM2_ESM.pdf]

Supplementary Figures

***KAT6A* mutations in Arboleda-Tham syndrome drive epigenetic regulation of posterior *HOXC* cluster**

Meghna Singh[1,2,3], Sarah J Spendlove[1,2,3,4], Angela Wei[1,2,3,4] ,Leroy M Bondhus[1,2,3] , Aileen A Nava[1,2,3], Francisca N. de L. Vitorino[5], Seth Amano[1,2,3], Jacob Lee[1,2,3], Gesenia Echeverria[1,2,3],Dianne Gomez[1,2,3], Benjamin A. Garcia[5], Valerie A. Arboleda[1,2,3,4]

## AFFILIATIONS

1 Department of Pathology & Laboratory Medicine, David Geffen School of Medicine, UCLA, Los Angeles, CA, USA
2 Department of Human Genetics, David Geffen School of Medicine, UCLA, Los Angeles, CA, USA
3 Department of Computational Medicine, David Geffen School of Medicine, UCLA, Los Angeles, CA, USA
4 Interdepartmental BioInformatics Program, UCLA
5 Department of Biochemistry and Molecular Biophysics, Washington University in St. Louis

**Corresponding Author:**
Valerie A. Arboleda
Associate Professor
Departments of Pathology & Laboratory Medicine, Human Genetics and Computational Medicine
David Geffen School of Medicine UCLA
615 Charles E. Young Drive South
Los Angeles, CA 90095
vaa2001@g.ucla.edu

Supplementary figure 1:

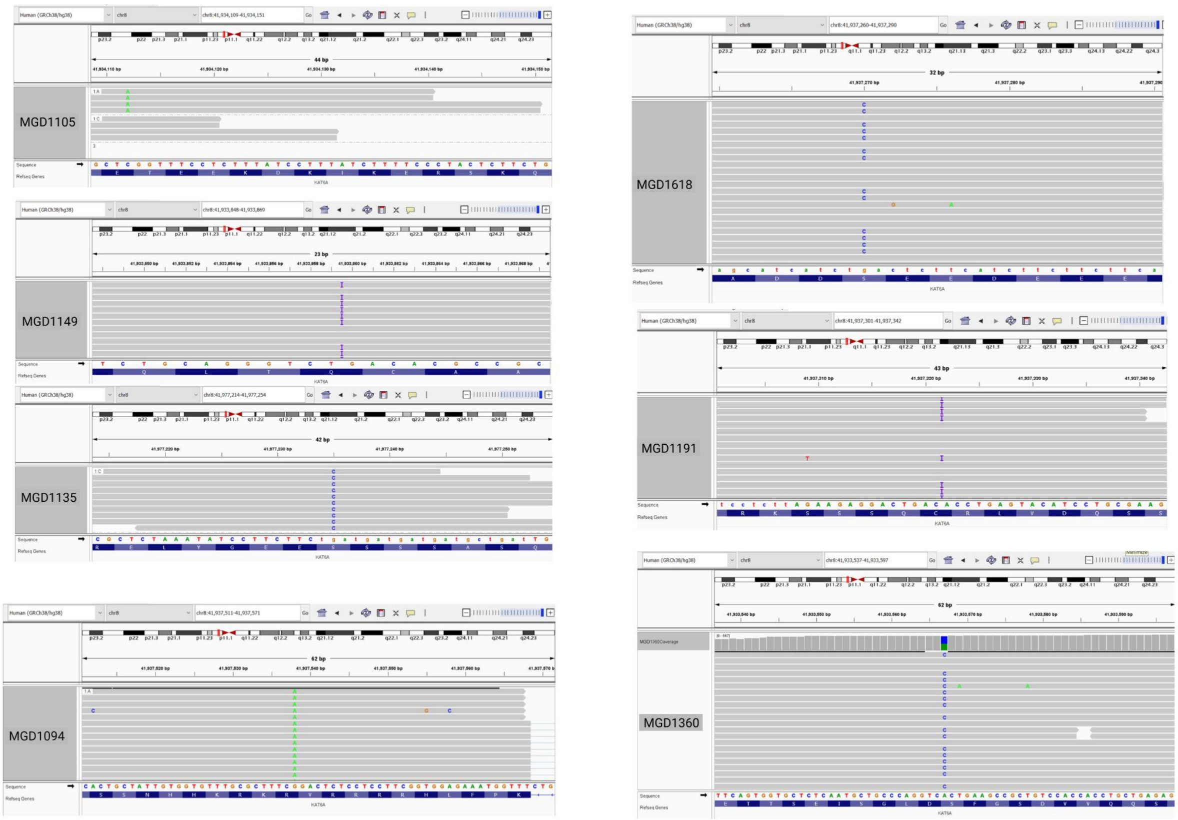


**Supplementary Figure 1: Genome track files confirming the ARTHS patient mutations.** All 7 ARTHS patient RNA-seq data was uploaded onto the IGV browser to confirm the mutations.

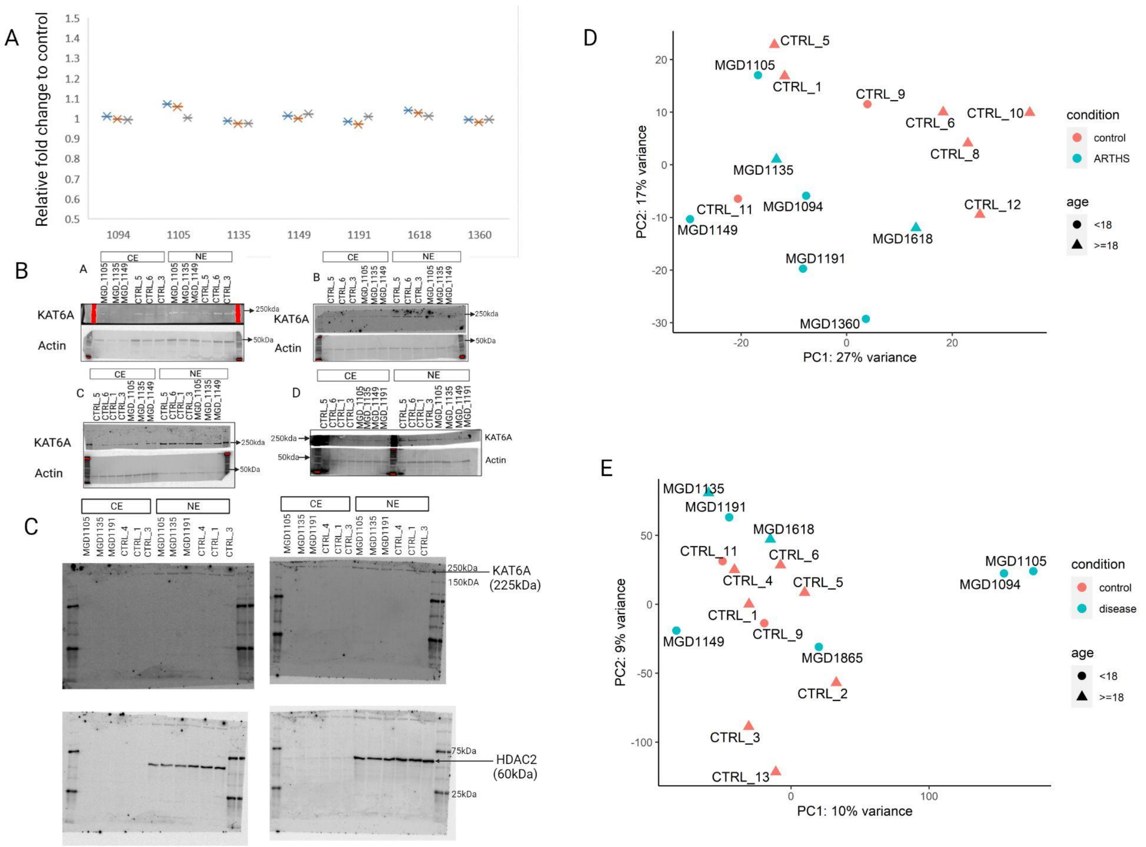


**Supplementary Figure 2**: A) RT-qPCR validation for the KAT6A transcripts does not show significant change in transcript levels. B) Four replicates of the western blot for KAT6A protein levels in cytoplasmic and nuclear fractions of cell pellets do not show significant changes in the ARTHS fibroblast samples as compared to the controls KAT6A (225kDA) Actin (40kDA). C) The blots here show the full blot images for the KAT6A (225kDA)(top blots) and HDAC2 (60kDA) (bottom blots). D)PCA plot for RNAseq data show that the ARTHS (green) and control (pink) samples mostly separate out on PCA 1 and 2 on the basis of sample type and age does not seem to drive the difference. E) PCA plot for the ATAC-seq dataset.

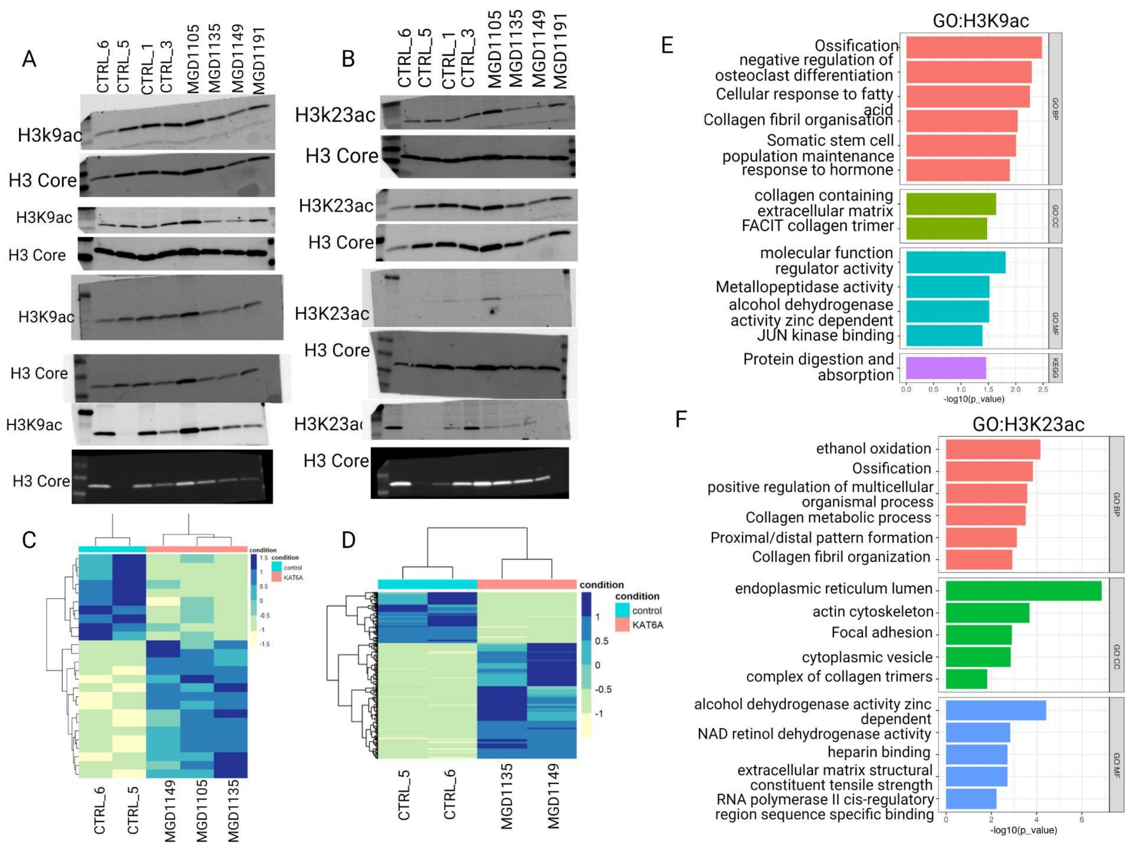


**Supplementary Figure 3: ARTHS mutations do not change global acetylation levels at H3K9 or H3K23.** A) Western blot images for the H3k9ac mark across 4 independent blots in a subset of ARTHS and control samples. These are quantified in Figure 4B. B) Western blot images for the H3K23ac mark across a subset of ARTHS and control samples. These are quantified in Figure 4C. Heatmap depicting differentially acetylated peaks from ChIP-seq data at the C) H3K9ac mark and D) H3K23ac mark. E) GO terms associated with differentially acetylated genes in the H3K9ac ChIPseq dataset F) GO terms associated with differentially acetylated genes in the H3K23ac ChIPseq dataset

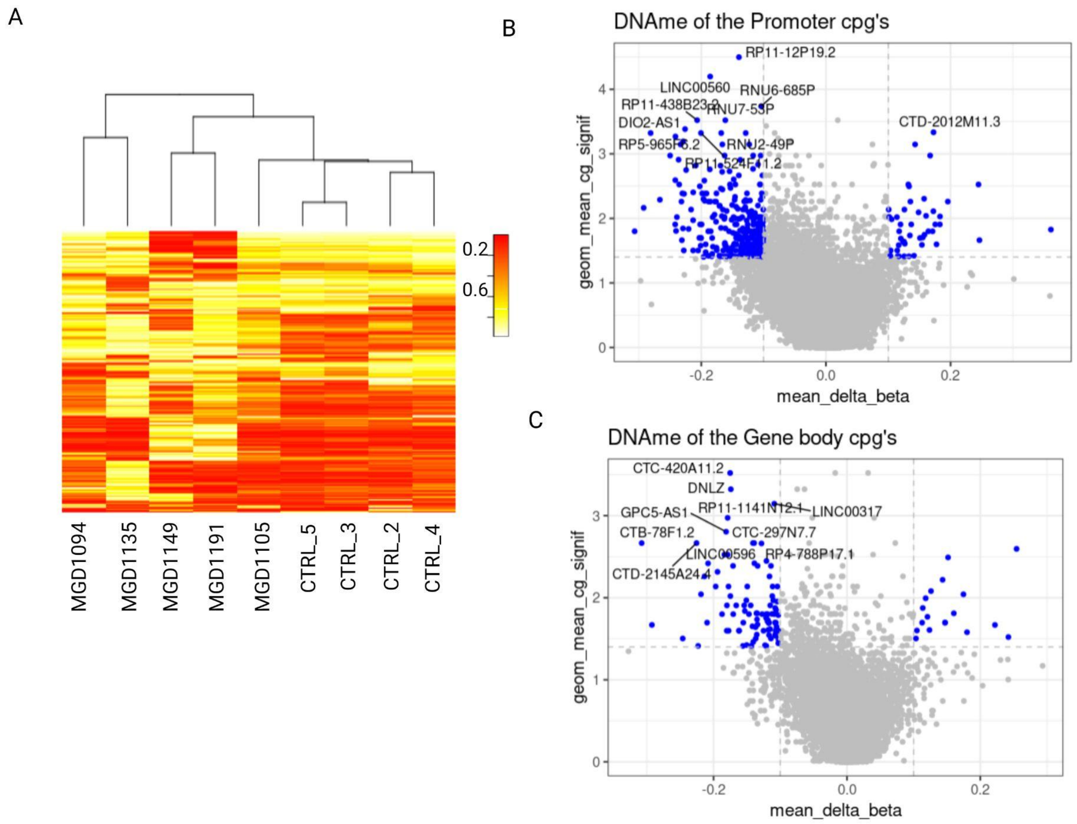


**Supplementary Figure 4:** CpG methylation profiling of ARTHS samples: A)Heatmap showing sample clustering and the levels of DNA methylation across ARTHS and control fibroblasts for the top 1000 most variable CpGs. B) Volcano plot showing promoter CpGs that are differentially methylated between ARTHS and control fibroblasts. Significantly different promoter CpGs are highlighted in blue. C) Volcano plot showing gene body CpGs that are differentially methylated between ARTHS and control fibroblasts. Significantly different genebody CpGs are highlighted in blue.

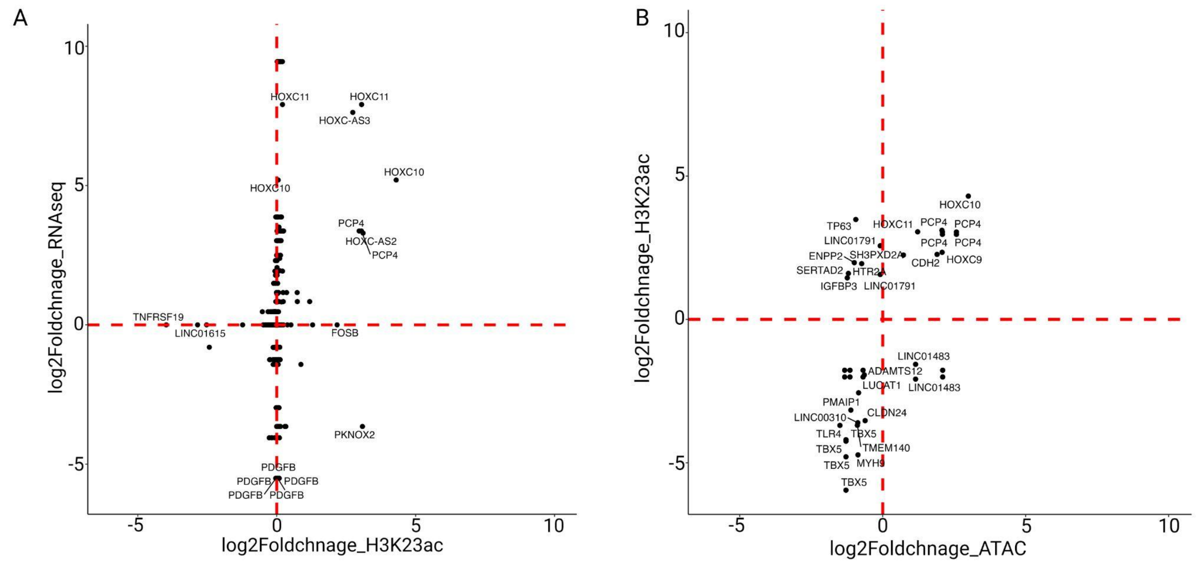


**Supplementary Figure 5:** Multi-omic data integration suggests that ARTHS *KAT6A* mutations regulate the expression of posterior *HOXC* cluster genes. A) Correlation between H3K23ac ChIPseq and RNAseq data. Our data highlights the expression change and H3K23 acetylation peaks on differentially expressed genes, including posterior *HOXC* genes. B) Correlation between H3K23ac ChIPseq and ATACseq data. It highlights increased H3K23 acetylation and increased chromatin accessibility over the posterior *HOXC* genes.
